# Inhalation of Ether

**Published:** 1847-05

**Authors:** 


### SELECTIONS

FROM

### AMERICAN AND FOREIGN JOURNALS.

---

*Inhalation of Ether. French Academy of Sciences.*—At a late meeting of the Academy of Sciences several communications were made relative to the effects of the inhalation of ether and some discussion ensued, in the course of which M. Majendie repeated his opinion that this new method of operating ought to be practised with the greatest possible caution and reserve. M. Majendie brought forward some further arguments in defence of his opinion, but they throw no new light upon the question. We shall proceed, therefore, to give an account of some experiments on the new method which were this day communicated to the Academy. M. Flourens stated that he had made experiments to ascertain the effect of the ether upon the spinal marrow. In the first place he caused the ether to be inhaled by a dog, which in a few minutes became perfectly insensible. He then laid bare the spinal marrow at a point of the dorsal region, and during the whole of this cruel operation the animal gave no sign of pain. He then divided the nerves of sensation, and still no pain was manifested. Lastly, he pricked, cut, and tore the spinal marrow itself, and the dog did not give the slightest symptom of suffering, and experienced no convulsion. His next experiment was on a fowl, and with the same results. When the effects of ether were dissipated, the spinal marrow resumed all its vitality. M. Serres announced that he had made several experiments upon animals, but with liquid ether, as he was desirous of ascertaining its effects upon the nervous system. He had laid bare the nerves of the thighs of several rabbits, and placed them in contact with a sponge dipped in ether. The results were as follow:—Sensibility was abolished in the nerve subjected to the action of ether at the points immediately in contact with it, and in all the radiations emerging from the nerve under that point; but the entire sense of feeling remained in the portion of the nerve above the point immerged in the ether. In order to take into account the action of the air, the following comparative experiment was made. Of two nerves which were laid bare, one was immerged in the ether, and the other was exposed simply to the action of the air. Five minutes afterwards the first was dead to all sensation, even on the application of pincers, the second retained all its sensibility and powers of contraction. Having thus ascertained the sedative power of the ether, M. Serres resolved to discover whether the sensibility could be restored by the immediate application of strychnine to the nerve which had been deprived of it. He applied the tincture of nux-vomica, strychnine, and the chlohydrate of strychnine, and all without effect. They produced neither sensibility to pain, nor restored the power of contraction.

A paper was read, giving an interesting account of some experiments made by M. Gruby, to ascertain the effects of ether on several animals, viz: 10 dogs, 4 rabbits, 2 mice, and 50 frogs. To intoxicate these animals required different periods of time, which are shown in the following table:—

| | *Minimum.* | *Maximum.* | *Duration.* |
|---|---|---|---|
| Frogs | 8 m. | 35 m. | 20 to 25 m. |
| Dogs | 50 | 40 | 12 to 30 |
| Mice | 2 | 4 | 4 to 8 |
| Rabbits | 2 | 8 | 6 to 12 |

In general, says M. Gruby, the duration of the intoxication appeared to depend on the intensity of the vapor, the prolongation of the aspiration, the rapidity and force of the inspiration, and the age of the animals. Beyond the term above fixed for respiration the animals died in a sleep. In young animals the phenomena were more prompt than in those which were full grown. M. Gruby found that the peripheric circulation, the pulsation of the heart, and the respiration diminish under the influence of the ethereal vapor, and that, if after the experiment the animal be exposed to the air, the number of pulsations of the heart and the inspiratory motion augment considerably, as does also the cutaneous circulation. Frogs which were wounded immediately before the experiment resisted the intoxicating efforts of the ether much longer than those which were uninjured. He ascertained also that the stagnation in the capillary vessels produced by the ether disappears before sensibility is again manifested, and that a frog deprived of its brain, and exposed to the vapors of ether, retains its sensibility and its powers of contraction much longer than one that has not been wounded. The experiments on dogs showed that they first lose their tactile peripheric sensibility, then voluntary motion, and finally the contractibility of the voluntary muscles. Dogs twenty days old lost their sensibility in three minutes, and died in eighteen to twenty minutes, under the effects of the ether; grown-up dogs lost their power of sensation in eight minutes, and died if the action of the ether was continued for forty-five minutes. The dogs recovered their sensibility and motion when they were exposed to the air, if the experiment with the ether was not prolonged beyond eighteen minutes for the young, and forty to forty-four minutes for adults. Young dogs which were apparently dead, having ceased to breathe, were brought to life by copious bleeding from the jugular vein. A young dog, which had already been under the effect of the ether and had recovered, was again exposed to it, and the same effect was produced in fifty seconds; but a dog which had been bled after the experiment, and was again exposed to the ether, resisted much longer than another dog which had not lost blood. In the cases where the experiment was carried to such a point as to produce death, M. Gruby found that the immediate cause of death was an accumulation of blood in the veins of the brain, those of the lungs, the liver, &c.

Several letters were received from surgeons giving account of successful operations, without pain, after the inhalation of ether.

A letter was received from M. E. Cotterau, informing the Academy that he has succeeded in producing an explosive powder from starch, by adopting nearly the same process as with cotton. This, however, was already known to chemists.—*Paris Press.* *D. W. Y.*

